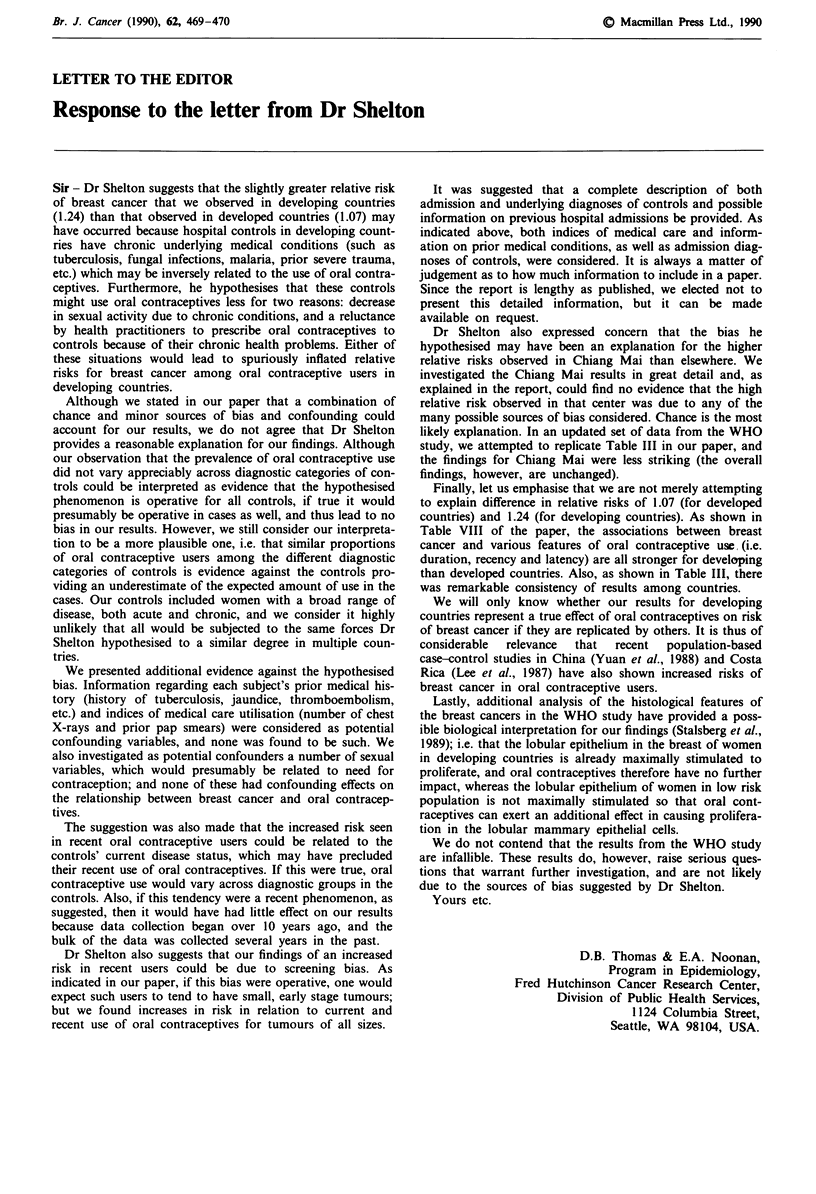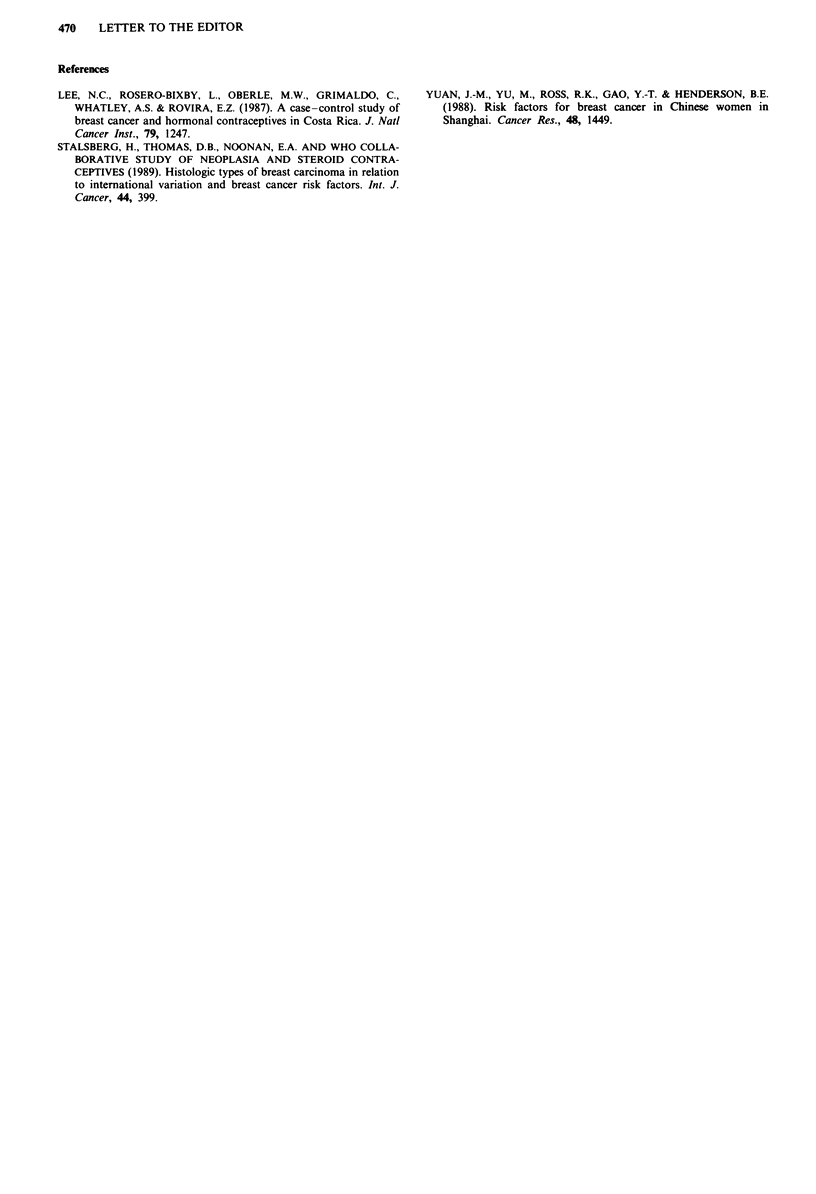# Response to the letter from Dr Shelton

**Published:** 1990-09

**Authors:** D.B. Thomas, E.A. Noonan


 © Macmillan Press Ltd., 1990

LETTER TO THE EDITOR

### Response to the letter from Dr Shelton

**Sir** – Dr Shelton suggests that the slightly greater relative risk of breast cancer that we observed in developing countries (1.24) than that observed in developed countries (1.07) may have occurred because hospital controls in developing countries have chronic underlying medical conditions (such as tuberculosis, fungal infections, malaria, prior severe trauma, etc.) which may be inversely related to the use of oral contraceptives. Furthermore, he hypothesises that these controls might use oral contraceptives less for two reasons: decrease in sexual activity due to chronic conditions, and a reluctance by health practitioners to prescribe oral contraceptives to controls because of their chronic health problems. Either of these situations would lead to spuriously inflated relative risks for breast cancer among oral contraceptive users in developing countries.

Although we stated in our paper that a combination of chance and minor sources of bias and confounding could account for our results, we do not agree that Dr Shelton provides a reasonable explanation for our findings. Although our observation that the prevalence of oral contraceptive use did not vary appreciably across diagnostic categories of controls could be interpreted as evidence that the hypothesised phenomenon is operative for all controls, if true it would presumably be operative in cases as well, and thus lead to no bias in our results. However, we still consider our interpretation to be a more plausible one, i.e. that similar proportions of oral contraceptive users among the different diagnostic categories of controls is evidence against the controls providing an underestimate of the expected amount of use in the cases. Our controls included women with a broad range of disease, both acute and chronic, and we consider it highly unlikely that all would be subjected to the same forces Dr Shelton hypothesised to a similar degree in multiple countries.

We presented additional evidence against the hypothesised bias. Information regarding each subject's prior medical history (history of tuberculosis, jaundice, thromboembolism, etc.) and indices of medical care utilisation (number of chest X-rays and prior pap smears) were considered as potential confounding variables, and none was found to be such. We also investigated as potential confounders a number of sexual variables, which would presumably be related to need for contraception; and none of these had confounding effects on the relationship between breast cancer and oral contraceptives.

The suggestion was also made that the increased risk seen in recent oral contraceptive users could be related to the controls' current disease status, which may have precluded their recent use of oral contraceptives. If this were true, oral contraceptive use would vary across diagnostic groups in the controls. Also, if this tendency were a recent phenomenon, as suggested, then it would have had little effect on our results because data collection began over 10 years ago, and the bulk of the data was collected several years in the past.

Dr Shelton also suggests that our findings of an increased risk in recent users could be due to screening bias. As indicated in our paper, if this bias were operative, one would expect such users to tend to have small, early stage tumours; but we found increases in risk in relation to current and recent use of oral contraceptives for tumours of all sizes.

It was suggested that a complete description of both admission and underlying diagnoses of controls and possible information on previous hospital admissions be provided. As indicated above, both indices of medical care and information on prior medical conditions, as well as admission diagnoses of controls, were considered. It is always a matter of judgement as to how much information to include in a paper. Since the report is lengthy as published, we elected not to present this detailed information, but it can be made available on request.

Dr Shelton also expressed concern that the bias he hypothesised may have been an explanation for the higher relative risks observed in Chiang Mai than elsewhere. We investigated the Chiang Mai results in great detail and, as explained in the report, could find no evidence that the high relative risk observed in that center was due to any of the many possible sources of bias considered. Chance is the most likely explanation. In an updated set of data from the WHO study, we attempted to replicate Table III in our paper, and the findings for Chiang Mai were less striking (the overall findings, however, are unchanged).

Finally, let us emphasise that we are not merely attempting to explain difference in relative risks of 1.07 (for developed countries) and 1.24 (for developing countries). As shown in Table VIII of the paper, the associations between breast cancer and various features of oral contraceptive use (i.e. duration, recency and latency) are all stronger for developing than developed countries. Also, as shown in Table III, there was remarkable consistency of results among countries.

We will only know whether our results for developing countries represent a true effect of oral contraceptives on risk of breast cancer if they are replicated by others. It is thus of considerable relevance that recent population-based case–control studies in China (Yuan *et al.*, 1988) and Costa Rica (Lee *et al.*, 1987) have also shown increased risks of breast cancer in oral contraceptive users.

Lastly, additional analysis of the histological features of the breast cancers in the WHO study have provided a possible biological interpretation for our findings (Stalsberg *et al.*, 1989); i.e. that the lobular epithelium in the breast of women in developing countries is already maximally stimulated to proliferate, and oral contraceptives therefore have no further impact, whereas the lobular epithelium of women in low risk population is not maximally stimulated so that oral contraceptives can exert an additional effect in causing proliferation in the lobular mammary epithelial cells.

We do not contend that the results from the WHO study are infallible. These results do, however, raise serious questions that warrant further investigation, and are not likely due to the sources of bias suggested by Dr Shelton.

Yours etc.

D.B. Thomas & E.A. Noonan,
Program in Epidemiology,
Fred Hutchinson Cancer Research Center,
Division of Public Health Services,
1124 Columbia Street,
Seattle, WA 98104, USA.

**References**

LEE, N.C., ROSERO-BIXBY, L., OBERLE, M.W., GRIMALDO, C., WHATLEY, A.S. & ROVIRA, E.Z. (1987). A case-control study of breast cancer and hormonal contraceptives in Costa Rica. *J. Natl Cancer Inst.*, **79**, 1247.

STALSBERG, H., THOMAS, D.B., NOONAN, E.A. AND WHO COLLABORATIVE STUDY OF NEOPLASIA AND STEROID CONTRACEPTIVES (1989). Histologic types of breast carcinoma in relation to international variation and breast cancer risk factors. *Int. J. Cancer*, **44**, 399.

YUAN, J.-M., YU, M., ROSS, R.K., GAO, Y.-T. & HENDERSON, B.E. (1988). Risk factors for breast cancer in Chinese women in Shanghai. *Cancer Res.*, **48**, 1449.